# In vitro antibiofilm activity of resveratrol against avian pathogenic *Escherichia coli*

**DOI:** 10.1186/s12917-021-02961-3

**Published:** 2021-07-20

**Authors:** Xiangchun Ruan, Xiaoling Deng, Meiling Tan, Chengbo Yu, Meishi Zhang, Ying Sun, Nuohao Jiang

**Affiliations:** 1 Laboratory of Veterinary Pharmacology and Toxicology, College of Animal Science and Technology, Anhui Agricultural University, 130 West Changjiang Road, Hefei, 230036 Anhui Province China; 2 Anhui Province Key Laboratory of Veterinary Pathobiology and Disease Control, Hefei, 230036 Anhui Province China

**Keywords:** Resveratrol, Avian pathogenic *Escherichia coli*, Biofilm, Antibiofilm activity

## Abstract

**Background:**

Avian pathogenic *Escherichia coli* (APEC) strains cause infectious diseases in poultry. Resveratrol is extracted from *Polygonum cuspidatum*, *Cassia tora Linn* and *Vitis vinifera*, and displays good antimicrobial activity. The present study aimed to investigate the antibiofilm effect of resveratrol on APEC in vitro. The minimum inhibitory concentration (MIC) of resveratrol and the antibiotic florfenicol toward APEC were detected using the broth microdilution method. Then, the effect of resveratrol on swimming and swarming motility was investigated using a semisolid medium culture method. Subsequently, the minimum biofilm inhibitory concentration (MBIC) and the biofilm eradication rate were evaluated using crystal violet staining. Finally, the antibiofilm activity of resveratrol was observed using scanning electron microscopy (SEM). Meanwhile, the effects of florfenicol combined with resveratrol against biofilm formation by APEC were evaluated using optical microscopy (OM) and a confocal laser scanning microscopy (CLSM).

**Results:**

The MICs of resveratrol and florfenicol toward APEC were 128 μg/mL and 64 μg/mL, respectively. The swimming and swarming motility abilities of APEC were inhibited in a resveratrol dose-dependent manner. Furthermore, resveratrol showed a significant inhibitory activity against APEC biofilm formation at concentrations above 1 μg/mL (*p* < 0.01). Meanwhile, the inhibitory effect of resveratrol at 32 μg/mL on biofilm formation was observed using SEM. The APEC biofilm was eradicated at 32 μg/mL of resveratrol combined with 64 μg/mL of florfenicol, which was observed using CLSM and OM. Florfenicol had a slight eradication effect of biofilm formation, whereas resveratrol had a strong biofilm eradication effect toward APEC.

**Conclusion:**

Resveratrol displayed good antibiofilm activity against APEC in vitro, including inhibition of swimming and swarming motility, biofilm formation, and could eradicate the biofilm.

**Supplementary Information:**

The online version contains supplementary material available at 10.1186/s12917-021-02961-3.

## Background

Avian pathogenic *Escherichia coli* (APEC) causes localized and systemic avian infections, which are responsible for considerable economic losses in the poultry industry [1]. Like many bacteria, APEC can live in two different forms, a planktonic state and a biofilm state [2]. APEC can form a single or multi-layer biofilm [3], which utilizes components of the extracellular polymeric substance (EPS). The main components of EPS include polysaccharides, proteins, nucleic acids, lipids, pili, and flagella [4]. Biofilm formation is one of the main mechanisms by which APEC develops drug resistance. It is difficult for antibiotics to invade the multilayer structure of the APEC biofilm and reach the cells to exert their effects [5]. Therefore, APEC in the form of biofilms often cause chronic, persistent, and repeated infections in the clinic, making treatment difficult [6, 7]. Biofilm formation has been linked to several reports of human and animal infectious diseases, as well as food source contamination [8, 9].

Biofilm formation can be divided into five stages: planktonic motility and reversible attachment; monolayer colony formation and permanent attachment; microcolony formation of flat structures; macrocolony formation of mushroom-like structures; and dispersion and reattachment [10]. Colonization and adhesion play an important role in biofilm formation [11]. Motility is necessary for colonization, adhesion, and infection of pathogens. *Escherichia coli* requires motility to colonize the host successfully and cause pathogenic infections. Flagella-mediated swimming and swarming motility are the main movement modes of *E. coli* [7]. To respond to, and cope with, a wide range of external conditions, *E. coli* must sense and respond quickly to external signals. *E. coli* flagellar motility is dependent upon the environment (pH and salt concentration), which are an essential part of the induction of adhesion on a host surface to enable biofilm formation [12]. Motility plays a critical role in the primary interaction with a surface and can promote *E. coli* biofilm development [13].

Natural products that inhibit and eradicate bacterial biofilms have been explored [14–16]. The natural product, resveratrol (trans - 3, 4, 5-trihydroxystilbene (C_14_H_12_O_3_)), exists in a variety of plants, such as *Polygonum cuspidatum*, *Cassia tora Linn*, and *Vitis vinifera*. It has a good health benefits, including antimicrobial, anti-inflammatory, anti-cancer, cardiovascular, and neuroprotective activities [17, 18]. The inhibitory activities of resveratrol toward bacteria [19, 20], fungi [21], and viruses [22, 23] have been reported. Meanwhile, the antibiofilm activity of resveratrol has been demonstrated via its inhibition of biofilm formation, eradication of biofilms [24], and inhibition of motility [25]. However, reports of its antibiofilm effect in vitro against clinically isolated APEC are limited. The present study aimed to investigate effects of resveratrol on the inhibition and eradication of APEC biofilms.

## Methods

### Chemicals and strain

Resveratrol was purchased from MedChemExpress (Monmouth Junction, NJ, USA). The resveratrol batch number was 58,706, and its purity (as assessed using liquid chromatography–mass spectrometry) was 99.70%. The antibiotic florfenicol was purchased from Hansyn Pharma (Jiangsu, China; 98% purity). The wild-type APEC strain was generously donated by Professor Li (The Livestock and Poultry Disease Diagnostic Centre of Anhui Agricultural University) and the intralaboratory serial number 11–28-2 was assigned to the strain. The strain was isolated aseptically from the livers of infected chickens.

### Minimum inhibitory concentration (MIC) determination and growth curve assay

The MICs toward APEC of resveratrol and florfenicol were detected using the broth microdilution method according to the American Committee for Clinical and Laboratory Standards Institute methods (CLSI). Briefly, 100 μL of resveratrol or florfenicol was added to the wells of 96-well U-bottomed plates using two-fold serial dilutions in Mueller-Hinton (MH) broth (pH 7.2–7.4). The final concentration ranged from 0.5 μg/ mL to 256 μg/ mL. APEC was cultured to the logarithmic growth stage. The culture was adjusted to OD_600_ = 0.1 (1 × 10^8^ CFU/mL), and then it was diluted 100 times to 1 × 10^6^ CFU/mL. Thereafter, each well was inoculated with 100 μL of the APEC inoculum (1 × 10^6^ CFU/mL) and cultured statically at 37 °C for 18–24 h. Meanwhile, MH broth as the negative control group and the APEC inoculum as the positive control were prepared, with three wells for each treatment as parallel samples.

To determine the growth curve, overnight cultures (200 μL) of APEC were added to 100 mL of fresh Luria-Bertani (LB) broth. The suspension was supplemented with resveratrol at a final concentration of 1/32 MIC, 1/16 MIC, 1/8 MIC, 1/4 MIC, and 1/2 MIC, respectively and incubated at 37 °C with constant shaking (200 rpm). At 0, 2, 4, 6, 8, 10, 12, 14, 16, 18, 20, 22, and 24 h, 3 mL of each sample was obtained, and the turbidity was measured at 600 nm using a spectrophotometer (UV-5100, Metash, Shanghai, China). The growth curve was constructed by plotting the absorbance versus the incubation time [26].

### Determining the maturation time of biofilm formation

Two hundred microliters of APEC culture (OD_600_ = 0.01) were added to the wells of a 96-well plate and incubated at 37 °C for 0.5, 1, 2, 3, 4, 5, 6, and 7 days, and the LB culture medium was changed every day. The blank control was LB nutrient broth, and each well was repeated four times as parallel samples. The planktonic bacteria were washed off using sterile phosphate-buffered saline (PBS). Each well was fixed with 200 μL of methanol for 15 min and dried naturally after removing the methanol. The formed biofilms were stained using 200 μL of crystal violet for 20 min. After removing the crystal violet, each well was washed three times with PBS. Then, 200 μL 33% acetic acid was added to the well and incubated for 30 min. The absorbance was measured at 570 nm using a Microplate Reader (Sunrise, Tecan, Switzerland) to determine the biomass of the biofilm. The definition of the different stages of APEC biofilm formation was based on description of the biofilm growth curve by Yu et al. [27].

### Motility assay

Swimming and swarming motility were investigated on semisolid culture medium in plates [26, 28]. LB medium was supplemented with 0.3% agar and 0.5% glucose for the swimming motility assay, and with 0.5% glucose and 0.5% agar for the swarming motility assay. Resveratrol was added to the LB medium at final concentrations of 1/32 MIC to 1/2 MIC. Thereafter, 2 μL of APEC culture (OD_600_ = 1) was placed on the center of the culture plates and incubated at 37 °C for 24 h. The tests were repeated three times.

### Minimum biofilm inhibitory concentration (MBIC) assay

The MBIC assay was performed as previously described with appropriate modifications [29, 30]. Briefly, the APEC culture was added with different concentrations of resveratrol (0, 1/128 MIC, 1/64 MIC, 1/32 MIC, 1/16 MIC, 1/8 MIC, 1/4 MIC, and 1/2 MIC, corresponding to 0, 1, 2, 4, 8, 16, 32, and 64 μg/mL, respectively). After incubation at 37 °C for 24 h, the planktonic cells were discarded, and the adherent cells were washed three times with PBS. Crystal violet staining was then performed as described in “Determining the maturation time of biofilm formation”. The absorbance was detected at 570 nm using a Microplate Reader. The inhibition rate was calculated in comparison with the positive control (0 μg/mL resveratrol) [31]. The inhibition rate = (OD _positive_ – OD _test_) / OD _positive_ *100%. Each experimental well was repeated four times.

### Biofilm inhibition analysis using scanning Electron microscopy (SEM)

The inhibitory effect of resveratrol on APEC biofilm formation was observed using SEM [32]. The slides (Φ = 10 mm), as adhesion carriers for the biofilm, were placed into 24-well plates. Then, 0.5 mL of APEC suspension (OD_600_ = 0.01) and 0.5 mL different concentrations of resveratrol (0, 1/32 MIC, 1/16 MIC, 1/8 MIC, 1/4 MIC, and 1/2 MIC) were added to the wells, which were incubated statically at 37 °C for 48 h. The slides were rinsed three times with PBS and fixed with 5% glutaraldehyde at 4 °C for 12 h. The samples were dehydrated with a graded series of ethanol/water mixtures (30, 50, 60, 70, 80, 90, and 100%) at each concentration for 20 min, and dehydrated in 100% acetone twice. Finally, the biofilm sample was assessed under a scanning electron microscope (S-4800, Hitachi, Tokyo, Japan) [33].

### Biofilm eradication analysis

#### Biofilm eradication rate

The APEC culture (OD_600_ = 0.01) was incubated in a 96-well plate at 37 °C for 36 h. The planktonic cells were removed, and the attached biofilm cells were washed three times with fresh LB broth. The attached biofilm cells were treated with florfenicol (MIC) and resveratrol (0, 1/32 MIC, 1/16 MIC, 1/8 MIC, 1/4 MIC, and 1/2 MIC, respectively). After incubation at 37 °C for 24 h, the planktonic cells were discarded, and the adherent cells were washed three times with PBS. Crystal violet staining was then performed as described in “Determining the maturation time of biofilm formation”. The absorbance was detected at 570 nm using a Microplate Reader and the eradication rate was calculated in comparison with the control [31]. Each well was repeated six times.

#### Optical microscopy (OM) observation

One milliliter of APEC suspension (OD_600_ = 0.01) was added to each well containing a sterile slide in a 24-well plate in advance and incubated statically at 37 °C for 36 h. After washing three times with fresh LB broth, florfenicol (MIC) and resveratrol (0, 1/32 MIC, 1/16 MIC, 1/8 MIC, 1/4 MIC, and 1/2 MIC, respectively) were added to the LB broth. After 24 h of treatment, the slides were washed with PBS, fixed with methanol for 15 min, and then stained with crystal violet. The slides were washed gently with PBS until the eluent was colorless. Finally, eradication of the biofilm was observed under an optical microscope (BA210, Motic, Xiamen, China) [34].

#### Confocal laser scanning microscopy (CLSM) observation

APEC biofilm culture and florfenicol and resveratrol treatment were the same as those described in the OM assay. The slides were washed gently with PBS and stained with the fluorescent dyes CFDA SE (5-(and 6)-Carboxyfluorescein diacetate, succinimidyl ester) and PI (Propidium Iodide). After incubation at 25 °C for 15 min, the slides were captured at excitation/emission wavelengths of 535/617 nm for PI, and 485/520 nm for CFDA SE using a confocal laser scanning microscope (FV1000, Olympus, Tokyo, Japan) [35].

### Statistical analysis

The data were expressed as the mean ± SD. The statistical differences among the different groups were compared using one-way analysis of variance (ANOVA), significant means were separated using Tukey’s Honest significant difference, and statistical significance was accepted at *p* < 0.05.

## Results

### MIC determination and growth curve construction

**Fig. 1:**
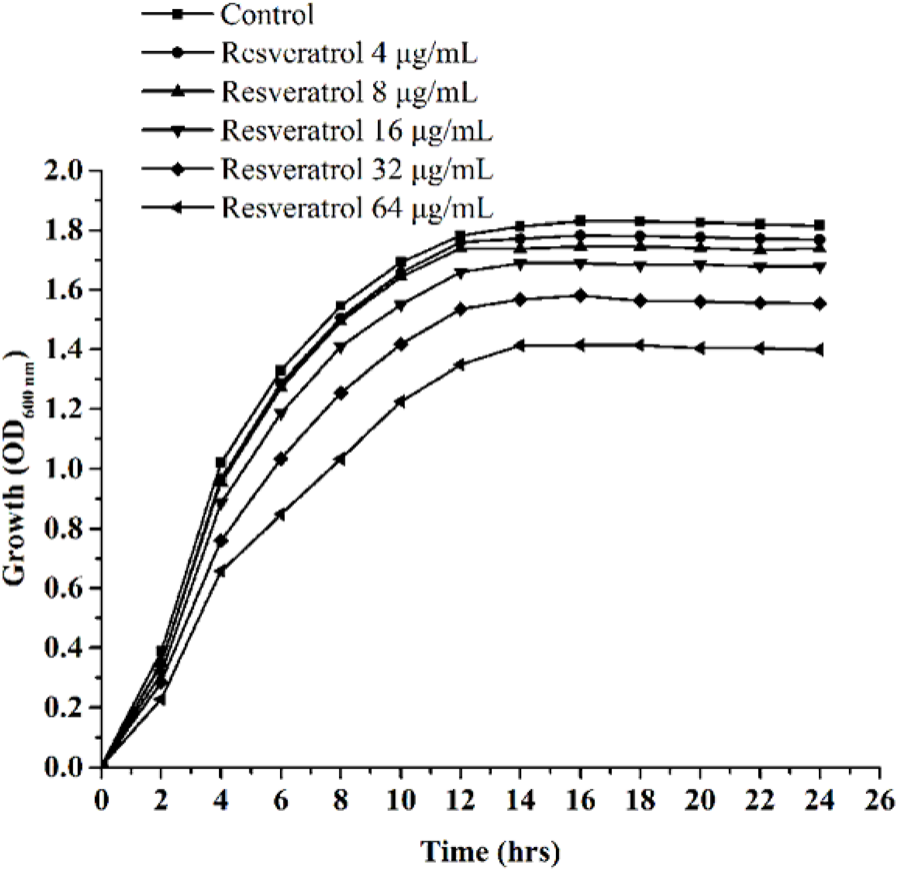
Growth curves of the avian pathogenic *Escherichia coli* strain under different concentrations of resveratrol

The MICs of resveratrol and florfenicol against planktonic APEC were 128 μg/mL and 64 μg/mL, respectively. Resveratrol had little effect on APEC growth at the 1/32 MIC, 1/16 MIC, 1/8 MIC, 1/4 MIC, and 1/2 MIC, which corresponded to concentrations of 4, 8, 16, 32, and 64 μg/mL, respectively (Fig. 1).

### Biofilm maturation time

**Fig. 2:**
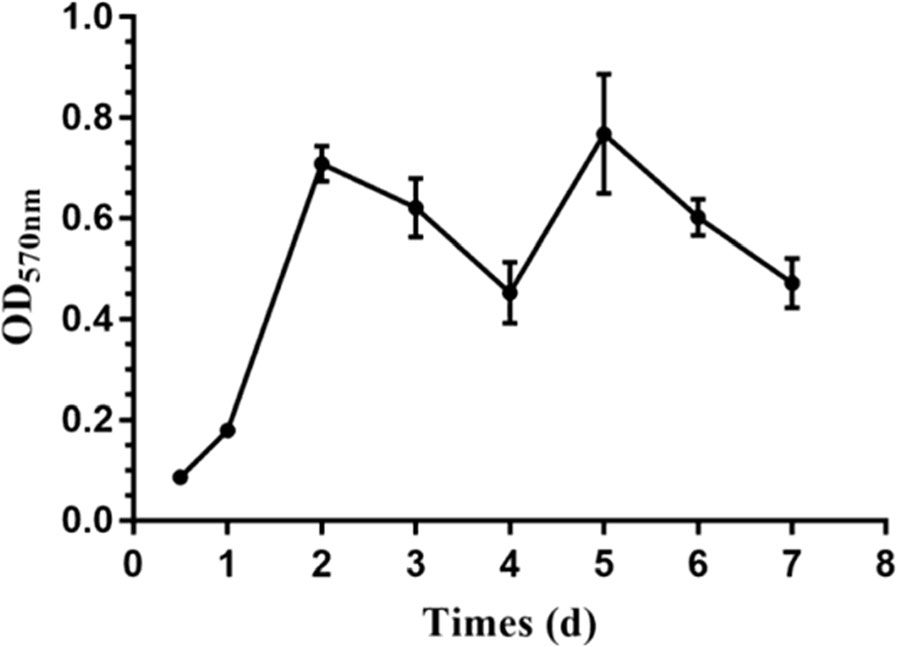
Curve showing the dynamics of biofilm formation by the avian pathogenic *Escherichia coli*

The biofilm formation of APEC is a dynamic process, comprising colonization, accumulation, maturation, shedding, and replantation stages. The complete process of biofilm formation took 4 days. The initial adhesion was completed after incubation for 12 h. The biofilm was formed at 1 day and matured at 2 days. The mature biofilm began to fall off from 2 to 4 d; and then the biofilm formation entered the next growth cycle (Fig. 2).

### Motility assay

**Fig. 3:**
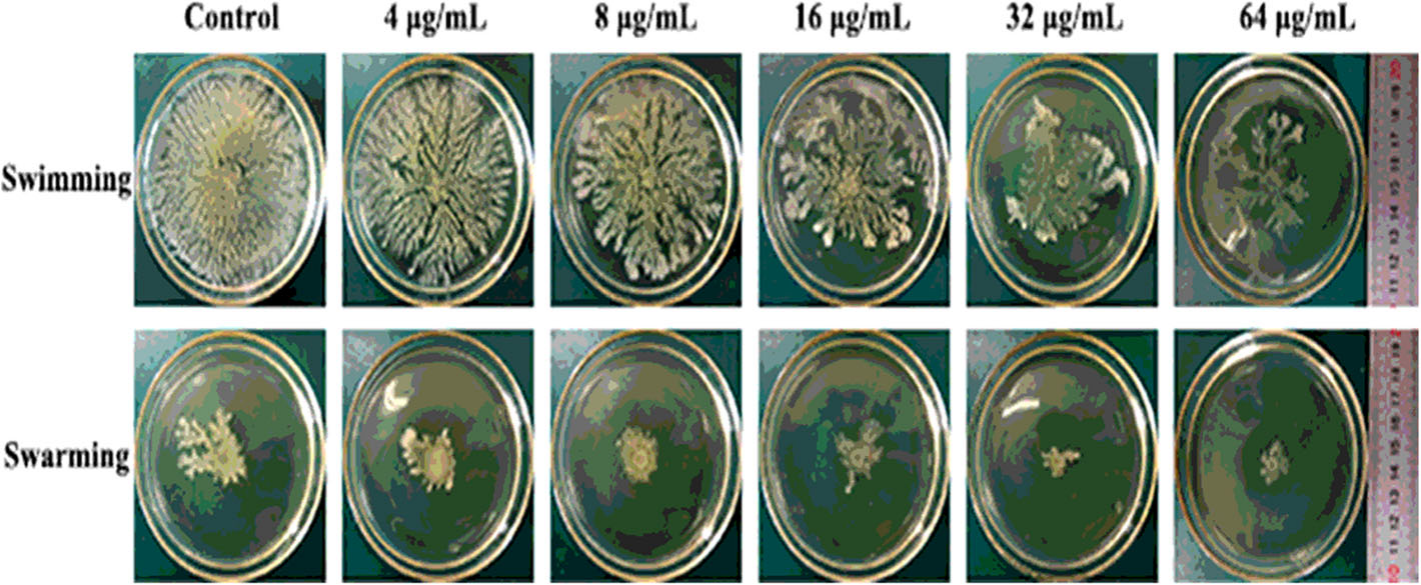
Inhibitory effect of resveratrol on the swimming and swarming motility

The effects of resveratrol on the swimming and swarming motility of APEC were evaluated (Fig. 3). With increasing resveratrol concentration, the swimming motility ability of APEC decreased gradually. The same result was found in the swarming motility assay. Thus, the inhibitory effect of resveratrol on APEC motility occurred in a dose-dependent manner.

of avian pathogenic *Escherichia coli.*

### MBIC assay

**Fig. 4:**
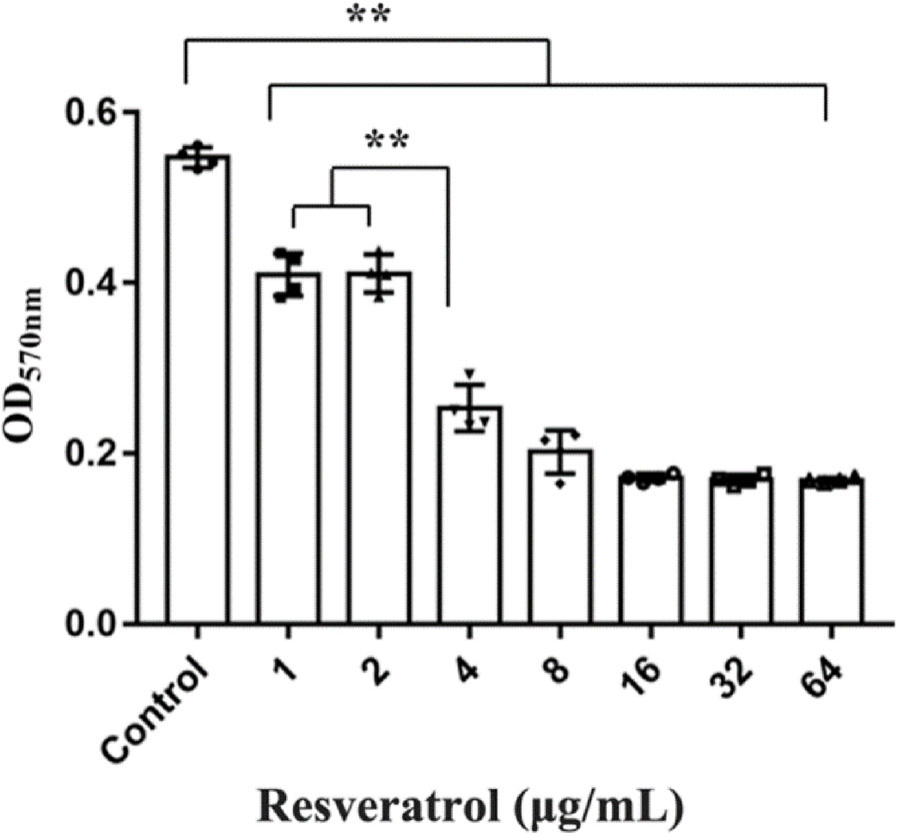
Inhibitory effect of resveratrol on avian pathogenic *Escherichia coli* biofilm formation (** *p* < 0.01)

Resveratrol had a strong inhibitory effect on the biofilm formation of APEC. Resveratrol inhibited biofilm formation significantly (*p* < 0.01) at 1 μg/mL (1/128 MIC), and the biofilm inhibition rate reached 31.66%. Biofilm formation was inhibited significantly at 4 μg/mL compared with that at 1 and 2 μg/mL of resveratrol (*p* < 0.01), and the inhibition rate reached 75.61%. The inhibitory effect of resveratrol on APEC biofilm formation also occurred in a dose-dependent manner (Fig. 4).

### Observation of biofilm inhibition using SEM

**Fig. 5:**
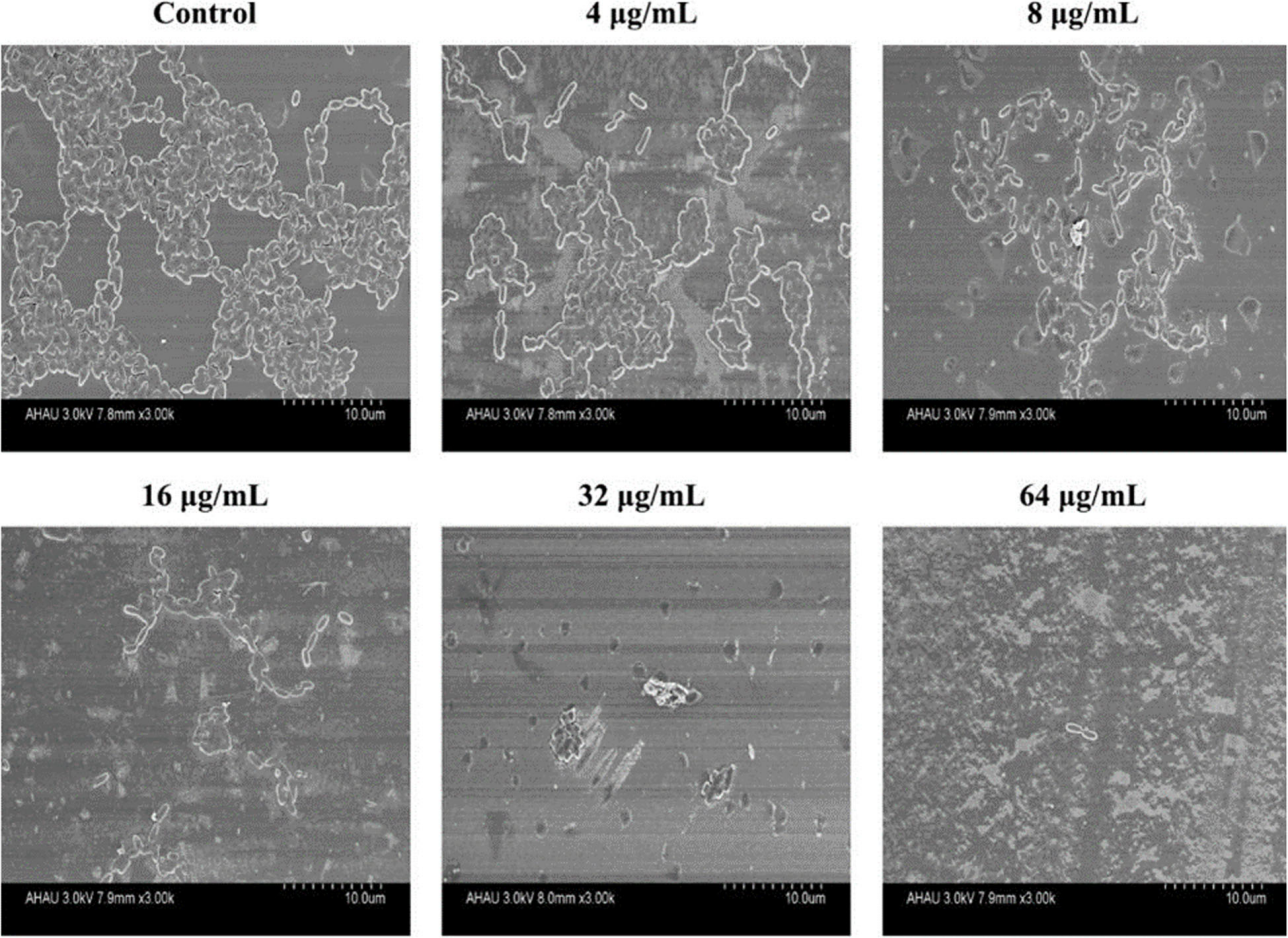
Inhibitory effect of resveratrol on avian pathogenic *Escherichia coli* biofilm formation observed using scanning electron microscopy

The inhibitory effect of resveratrol on APEC biofilm formation was observed using SEM. The APEC cells adhered tightly and formed a dense biofilm on the control slides. The biofilm decreased and became sparse on the slides after resveratrol treatment at 4 μg/mL. When the resveratrol concentration increased from 4 μg/mL to 16 μg/mL, APEC biofilm formation was inhibited increasingly on the slides. At a resveratrol concentration of 32 μg/mL, only a few APECs adhered to the slides, and at 64 μg/mL, scattered or individual APEC cells were observed on the slides (Fig. 5).

### Biofilm eradication analysis

**Fig. 6:**
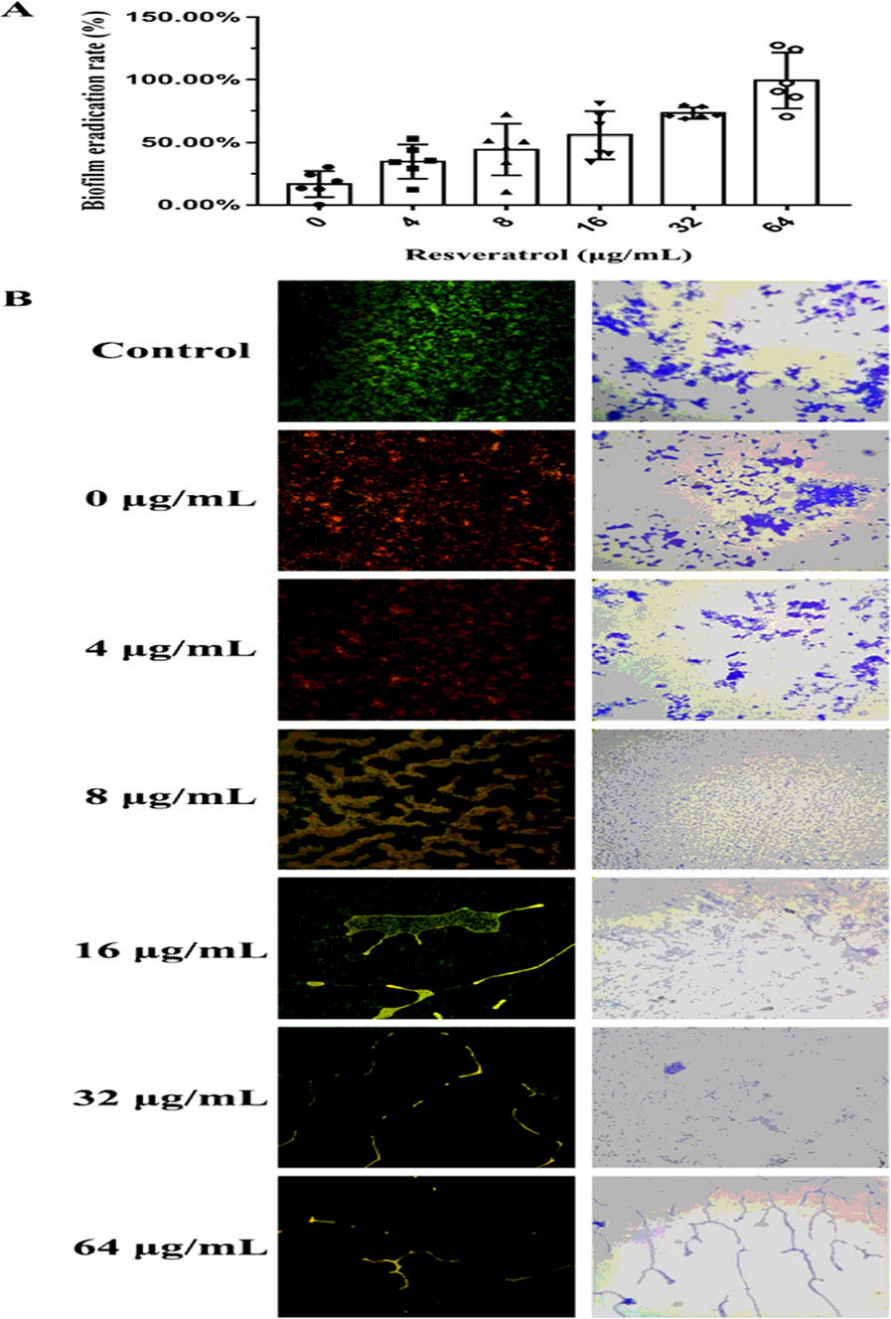
The eradication effect of resveratrol on avian pathogenic *Escherichia coli* biofilm

In the absence of resveratrol (0 μg/mL), the eradication rate by florfenicol on the biofilm was 16.74% at 64 μg/mL (MIC). Meanwhile, in presence of florfenicol at 64 μg/mL, the eradication rate of biofilm increased gradually with increasing resveratrol concentration. When the concentration of resveratrol reached 16 μg/mL and 32 μg/mL, the eradication rates of biofilm were 55 and 73%, respectively. Furthermore, the eradication rate of biofilm exceeded 99% at a resveratrol concentration of 64 μg/mL (Fig. 6A). These results revealed that florfenicol had a slight biofilm eradication effect at 64 μg/mL. Comparatively, resveratrol at 32 μg/mL showed a strong biofilm eradication effect toward APEC.

Under CLSM observation, viable APEC cells with intact membranes were stained fluorescent green, whereas dead cells with damaged membranes were stained fluorescent red, and the coexistence of living and dead cells appeared yellow. A dense biofilm formed and living APEC cells were visible on the slides of the control (Fig. 6B, Left, 400×). The majority of APEC cells located in the outermost layer of the biofilm were dead after treatment with florfenicol alone at 64 μg/mL. However, a tight and dense biofilm still existed on the slides. This indicated that florfenicol could not destroy the APEC biofilm (Fig. 6B, Left, 400×). As the resveratrol concentration increased from 4 μg/mL to 16 μg/mL, the biofilm formed on the slides decreased gradually. The dense biofilm was destroyed and the number of large pieces of the biofilm decreased. Most of the APEC biofilm on the slides was eradicated after resveratrol treatment at 32 μg/mL. Therefore, resveratrol has a strong eradication effect on the biofilm formed by APEC. Similar results were obtained using OM observation (Fig. 6B, Right, 100×).

## Discussion

Clinical bacterial infectious diseases are very common, among which about 80% are related to bacterial biofilms [36]. A biofilm formed during infection is rarely eradicated by the host defense mechanisms. Bacterial adhesion and colonization play an important role in the process of biofilm formation. The pathogenicity of APEC is enhanced during the formation of a biofilm, which is caused by adhesion and colonization [37]. Some researchers believe that bacterial biofilms have nothing to do with pathogenicity in vivo [38]. However, the majority opinion suggests that bacterial biofilms are associated with pathogenicity in vivo. Most clinically isolated APECs have a strong or medium biofilm forming ability, which might be explain their high pathogenicity and difficult treatment [39]. It was reported that compared with planktonic cells, bacteria in the state of a biofilm can resist the host’s immune defense [40]. Therefore, inhibiting the formation of APEC biofilms is of great significance in the clinical study of diseases caused by APEC.

Antibiotic therapy typically alleviates the symptoms caused by planktonic bacteria, but fails to kill bacteria in biofilms [6]. During aggravation of bacterial resistance, traditional Chinese medicines or their extracts have attracted attention. Plant-derived drugs have been applied widely in infection therapy because they are effective, safe, have low toxicity, and induce lower levels of acquired resistance [41–43]. Some compounds extracted from plants possess antibacterial and antibiofilm activities [44, 45]. Resveratrol, a natural product extracted from *Polygonum cuspidatum, Cassia tora Linn*, or *Vitis vinifera*, can inhibit and eradicate bacterial biofilms. The main antibiofilm activities of resveratrol comprise inhibiting the quorum sensing system, metabolism, and bacterial motility (chemotaxis) [46–49]. In the present study, resveratrol inhibited the formation of biofilms and destroyed formed biofilms in a dose-dependent manner. This was consistent with the results of resveratrol inhibition of biofilm formation by *Fusobacterium nucleatum* [47]. Resveratrol shows a strong antibiofilm activity toward APEC; thus, the mechanism of action of resveratrol on APEC biofilms should be further investigated.

Biofilm formation is affected by environmental factors, the contact surface, phylogeny, and two-component systems. Environmental factors include temperature, pH, osmotic pressure, ions, nutrients, and other factors derived from the biological environment. APEC biofilm formation seems to be mostly limited to nutrient depleted media; whereas, avian fecal *Escherichia coli* (AFEC) are able to form biofilms in both nutrient depleted and replete media [50]. Adhesion and colonization play an important role in biofilm formation [11]. Motility is necessary for the process of colonization, adhesion, and infection by pathogens. *E. coli* motility includes swimming and swarming. The results of the present study showed that resveratrol inhibited the swimming and swarming motility of APEC in a dose-dependent manner. These results were consistent with those of previous reports [51, 52]. In addition, resveratrol was effective in preventing APEC colonization and adhesion on slide surfaces, which was verified using OM, SEM, and CLSM observations. It was speculated that resveratrol decreased the adhesion of APEC cells by inhibiting their swimming and swarming motility abilities, which in turn would inhibit biofilm formation [53, 54].

Crystal violet staining was used to identify the antibiofilm effect of resveratrol on APEC in this study. This technique is semiquantitative, simple, fast, and widely used to detect bacterial biofilms in the laboratory [31, 35]. However, the absorbance readings could only be used to determinate biofilm formation accurately when the readings were taken from a homogeneous bacterial suspension [55].. It is possible that the cells could be unevenly packed or irregularly stuck to a surface, which might affect the absorbance readings. Therefore, the antibiofilm effect of resveratrol on APEC was observed using OM, SEM, and CLSM methods, which could compensate for the limitations of crystal violet staining [33–35].

The combined use of a biofilm inhibitor and an antibiotic causes a certain degree of damage to the biofilm and increases the opportunity for the antibiotic to contact the bacteria in the biofilm, resulting in synergistic antibacterial activity and the destruction of the biofilm [56, 57]. APEC biofilms on the slides were not destroyed after treatment with florfenicol alone at the MIC (64 μg/mL). This might be related to resistance to florfenicol. Meanwhile, as the resveratrol concentration increased from 4 μg/mL to 16 μg/mL, the APEC biofilm decreased gradually and became sparsely distributed on the slides. There were no large pieces of biofilm on the slides, and the biofilm was destroyed and fell off under treatment with resveratrol at 32 μg/mL and 64 μg/mL. Resveratrol at 32 μg/mL inhibited and eradicated APEC biofilms, as observed using SEM, CLSM, and OM. These results are consistent with those obtained by crystal violet staining. The results of motility assay indicated that resveratrol inhibited adhesion and colonization of APEC by inhibiting swimming and swarming motility. Therefore, resveratrol was proven to have a strong eradicating effect on APEC biofilms in vitro. These promising results will lead to further preclinical and in vivo testing of the antibiofilm effects of resveratrol.

## Conclusions

The natural product resveratrol inhibited the formation of APEC biofilms and showed a strong in vitro eradication effect toward APEC biofilms. By contrast, the effect of florfenicol alone on the eradication APEC biofilms was negligible. Furthermore, there was no obviously improved effect of the combination of florfenicol and resveratrol on the eradication of the APEC biofilm. The eradication effect toward biofilms increased gradually with increasing resveratrol concentrations. Interestingly, we also found that resveratrol inhibited the APEC swimming and swarming motility, which might represent the mechanism by which resveratrol inhibits APEC biofilm formation; however, this requires further study. The results suggested that resveratrol could be applied to inhibit and eradicate APEC biofilms and provides a new direction for the prevention and control of diseases caused by APEC.

## Supplementary Information

**Additional file 1.**

## Data Availability

All data generated or analyzed during this study are included in this published article and its supplementary information files.

## Abbreviations

APEC: Avian pathogenic *Escherichia coli*
CFDA SE: 5-(and 6)-Carboxyfluorescein diacetate, succinimidyl ester
CLSI: Clinical and Laboratory Standards Institute
CLSM: Confocal laser scanning microscopy
MIC: Minimum inhibitory concentration
MBIC: Minimum biofilm inhibitory concentration
OM: Optical microscopy
PI: Propidium Iodide
SEM: Scanning electron microscopy.

## Declarations

All authors have read and approved this version of the article, and due care has been taken to ensure the integrity of the work.
